# Supplementary material for: Production of deoxycholic acid by low-abundant microbial species is associated with impaired glucose metabolism
Source: Nat Commun. 2024 May 20;15:4276. doi: 10.1038/s41467-024-48543-3 (PMC11106306; doi:10.1038/s41467-024-48543-3)
Supplement: Supplementary file 1 — Supplementary Information [file 41467_2024_48543_MOESM1_ESM.pdf]

Supplementary Table 1. Bacterial strains included in the synthetic communities. Bacterial strains included in the base community (*Bacteroides caccae* ATCC 43185, *Bacteroides vulgatus* ATCC 8482, *Eubacterium rectale* ATCC 33656, *Bacteroides thetaiotaomicron* VPI-5482, *Bacteroides uniformis* ATCC 8492, *Parabacteroides distasonis* ATCC 8503, *Bacteroides ovatus* ATCC 8483, *Ruminococcus torques* ATCC 27756 and *Dorea longicatena* DSM 13814) and *Clostridium scindens* ATCC 35704. The genes, forward and reverse primers, and probes that were used to quantify each specific bacterial strain in the digital droplet PCR are presented. *TilS*, tRNAIle-lysidine synthase; *BaiCD*, bile acid-inducible gene CD.

Target Strain	Gene	Forward Primer/Reverse Primer	Probe
Bacteroides uniformis ATCC 8492	*tilS*	CCTGGGATACACATGCGAAG/<br>TGTTGAAGTGGACGGTGTG	/56-FAM/CAGATGGAA/ZEN/GTTGCAATGGGCGG/3IABkFQ/
Bacteroides vulgatus ATCC 8482	*tilS*	CGGGACGATAGTGTGGAAAC/<br>AAAATATCCTGACGGCTCACC	/56-FAM/CGCGGGAAT/Zen/CAAT GGCTTGAAAGG/3IABkFQ/
Bacteroides thetaiotaomicron VPI 5482	*tilS*	GATGCTCTACTGGACGAACC/<br>TGCCGAATTGAATCCCAGTG	/56-FAM/CCGCTCCCG/Zen/AAGCTTTCTTGTTTG/3IABkFQ/
Bacteroides caccae ATCC 43185	*tilS*	GTGGTATGCAATCGGATGTTG/<br>AGG TTA TCA CTG CGA AGC C	/56-FAM/TATTTCAGG/Zen/CAGAGTTGGCGGACA/3IABkFQ/
Bacteroides ovatus ATCC 8483	*tilS*	ATCATTACGGCAGAAGGTATCAG/<br>GGTGATATCTTTGATTTGTGCGG	/56-FAM/TCTGTGATT/Zen/TGGT AAA GGA ACC GGC A/3IABkFQ/
Eubacterium rectale ATCC 33656	*tilS*	ATCTGAAGGAAAACGGACAGG/<br>AGCTTGTTTCTGGAGTAGGC	/56-FAM/TGTGTCAGT/ZEN/GTTTGTGGCATCGGT/3IABkFQ/
Parabacteroides distasonis ATCC 8503	*tilS*	GGGCCGATGGATTTATCTCC/<br>CGTAAGCCGTGAGGATATCTTG	/56-FAM/CAT CCG TTT /ZEN/GAA TGT GTT GCC GCT /3IABkFQ/
Ruminococcus torques ATCC 27756	*tilS*	AGAGGACATATGACGGGATAGAG/<br>CCACAAAACGCAGTTCCAAC	/56-FAM/ATAAGGCAA/ZEN/ACACAAAGCAGGGCG/3IABkFQ/
Dorea longicatena DSM 13814	*tilS*	ATCTACATCCGCACTTTCTCC/<br>TGTAATTGCAGGCGTATCGG	/56-FAM/ATATGCACT/ZEN/ACCGTCAACCCAAACCG/3IABkFQ/
Clostridium scindens ATCC 35704	*baiCD*	GAACAGACCATGACAGAGGAAG/<br>AGGTTGAATGGCGGTACTTC	/56-FAM/CAGAACTG/Zen/GCGTGGATGT GGC/3IABkFQ/

Supplementary Figure 1

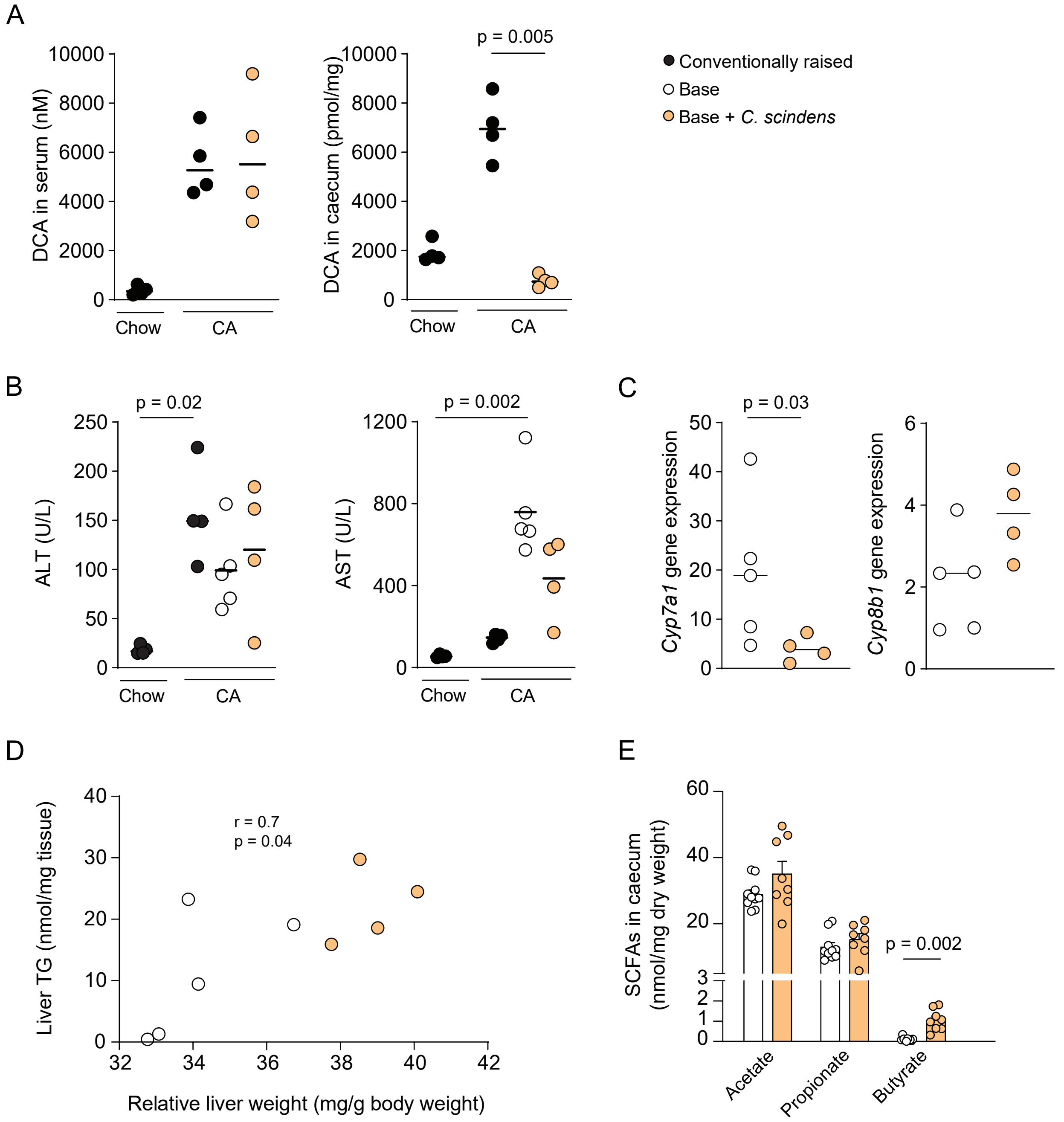

Supplementary Figure 1. DCA levels in serum and caecum, liver transaminases, hepatic gene expression and triglycerides, and caecal short chain fatty acids. (A) DCA levels in serum and caecum from conventionally raised mice and mice colonized with bacterial communities (base community or base community + *C. scindens*.) (B) Liver transaminases, ALT and AST, in conventionally raised mice and mice colonized with bacterial communities (C) Hepatic gene expression of *Cyp7a1* and *Cyp8b1* in mice colonized with bacterial communities assessed by qPCR. (D) Spearman's correlation analysis between liver triglycerides (TG) and relative liver weight in the colonized mice. (E) Levels of short chain fatty acids in mmol/mg dry weight of caecal content from the colonized mice. Black color respresent conventionally raised mice (n=4) for A-B; white color represent mice colonized with base community (n=5) for A-D and (n=10) for E; orange color represent mice colonized with base community + *C. scindens* (n=4) for A-D and (n=8) for E; P-values indicate significant differences using Kruskal-Wallis with Dunn's multiple comparison test when more than two groups are compared (A and B) or two-tailed Mann-Whitney when two groups are compared (C and E).

Supplementary Figure 2

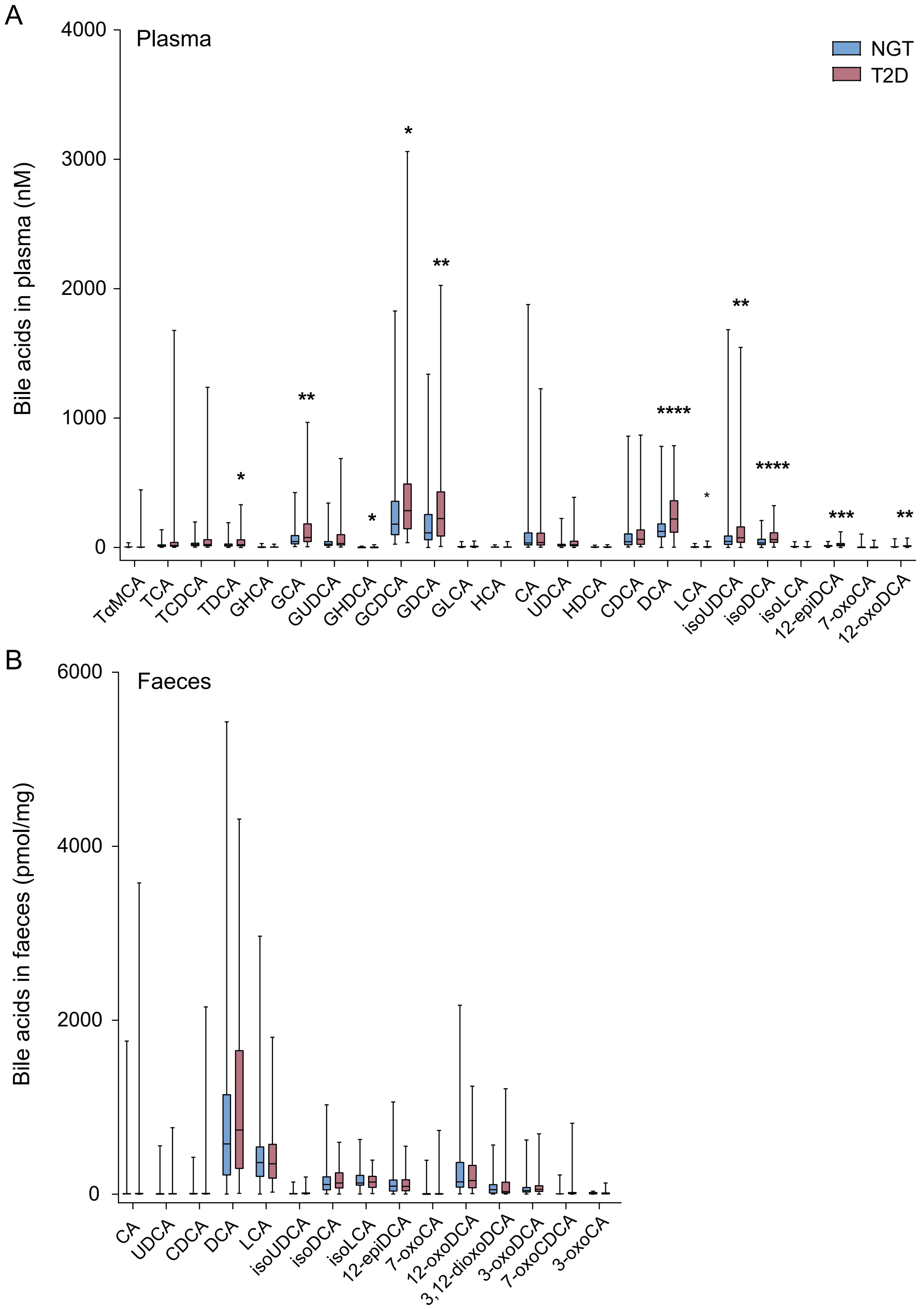

Supplementary Figure 2. Bile acid profiles in plasma and faeces of NGT and T2D individuals. (A-B) Bile acids in plasma presented in nM (A) and faeces presented in pmol/mg (B) of NGT and T2D individuals analyzed by UPLC-MS/MS. Levels of DCA, isoDCA, 12βDCA in plasma and DCA in faeces are also presented in Figure 3B-D and Figure 3F. Data are presented as box-and-whisker plots where whiskers represent min and max, n=100 samples/group;* P<0.05, ** P <0.01, *** P <0.001, *** P <0.0001 indicate differences in bile acid levels between NGT (blue color) and T2D (pink color) analyzed by Mann-Whitney using Benjamini and Hochberg adjusted p-values. CA, cholic acid; CDCA, chenodeoxycholic acid; DCA, deoxycholic acid; HCA, hyocholic acid; HDCA, hyodeoxycholic acid; GCA, glyco-cholic acid; GCDCA, glyco-chenodeoxycholic acid; GDCA, glyco-deoxycholic acid; GHCA, glyco-hyocholic acid; GHDCA, glyco-hyodeoxycholic acid; GLCA; glyco-lithocholic acid; GUDCA. glyco-ursodeoxycholic acid; isoDCA, iso-deoxycholic acid (3β-deoxycholic acid); isoLCA, iso-lithocholic acid (3β-lithocholic acid); isoUDCA, iso-ursodeoxycholic acid (3β-ursodeoxycholic acid); LCA, lithocholic acid; NGT, normal glucose tolerance; T2D, Type 2 diabetes; TCA, tauro-cholic acid; TCDCA, tauro-chenodeoxycholic acid; TDCA, tauro-deoxycholic acid; TαMCA, tauro-alpha-muricholic acid; UDCA, ursodeoxycholic acid; 3-oxoCA, 3-oxocholic acid; 3-oxoDCA, 3-oxodeoxycholic acid; 3,12-dioxoDCA; 3,12-dioxodeoxycholic acid; 7-oxoCA; 7-oxocholic acid; 7-oxoCDCA, 7-oxochenodeoxycholic acid; 12-epiDCA, 12-epideoxycholic acid (12β-deoxycholic acid); 12-oxoDCA, 12-oxodeoxycholic acid.
