# Supplementary material for: Production of deoxycholic acid by low-abundant microbial species is associated with impaired glucose metabolism
Source: Nat Commun. 2024 May 20;15:4276. doi: 10.1038/s41467-024-48543-3 (PMC11106306; doi:10.1038/s41467-024-48543-3)
Supplement: Supplementary file 3 — Description of Additional Supplementary Files [file 41467_2024_48543_MOESM3_ESM.docx]

**Inventory of Supporting Information**

File name: Supplementary Data 1

Description: Bile acid profiles in plasma in NGT and T2D individuals and correlation analyses between bile acids and clinical parameters.

File name: Supplementary Data 2

Description: Bile acid profiles in faeces in NGT and T2D individuals and correlation analyses between bile acids and clinical parameters (separate file).
